# Clinical Characteristics and Outcomes of Acute Lung Injury Caused by Transcatheter Arterial Chemoembolization for Hepatocellular Carcinoma: A Retrospective Cohort Study from a Single Institution in China

**DOI:** 10.1155/2019/4307651

**Published:** 2019-11-26

**Authors:** Liang-jie Fang, Lu-yan Chen, Jun-hui Sun, Jian-ying Zhou

**Affiliations:** ^1^Department of Respiratory Medicine, First Affiliated Hospital, School of Medicine, Zhejiang University, Hangzhou 310003, China; ^2^Department of Surgery, First Affiliated Hospital, School of Medicine, Zhejiang University, Hangzhou 310003, China

## Abstract

**Background:**

Acute lung injury (ALI) is a rare but life-threatening pulmonary complication of transcatheter arterial chemoembolization (TACE) for hepatocellular carcinoma (HCC). The aim of this study was to characterize the common risk factors, clinical features, imaging findings, treatments, and outcomes of acute lung injury caused by TACE.

**Methods:**

A retrospective study was performed on all TACE-associated ALI cases that were diagnosed at authors' hospital from January 2015 to June 2018.

**Results:**

The study included 14 ALI cases where the mean age of patients was 60.9 ± 11.7 years (range 41-82 years), with a mean onset time of 2.4 ± 1.6 d after TACE. Of the 14 patients, 8 patients (57.1%) developed acute respiratory distress syndrome (ARDS). 7 patients (50%) had underlying chronic respiratory disease and hepatic arteriovenous fistula was detected in 6 patients (42.6%), both of which were significantly higher than control group (*P* < 0.05). Dyspnea (92.9%) was the most common symptoms. Pleural effusion (64.3%), diffuse pulmonary infiltration (42.9%), and accumulation of Lipiodol in lung field (42.9%) were frequent radiologic abnormalities. 11 patients (78.6%) achieved remission after treatment, and the 30-day mortality rate was approximately 21.4%. Patient's median survival time after the development of ALI was merely 4.3 months, which was obviously worse than control group (4.3 months vs. 13.5 months, *P* < 0.05).

**Conclusion:**

This study illustrates that TACE-associated ALI is a rare pulmonary complication with a high mortality rate. We infer that pulmonary Lipiodol embolization might be one of the main causes of TACE-associated ALI. Thus, HCC patients who are at high risk should be closely evaluated and monitored during TACE to avoid such potentially fatal complication.

## 1. Introduction

Hepatocellular carcinoma (HCC), the most common primary hepatic malignancy, is a leading cause of cancer-related death in the world, and more than 80% of the cases occur in Asia due to the prevalence of chronic hepatitis [1, 2]. Transcatheter arterial chemoembolization (TACE), a palliative therapy, first reported in the 1970s, has been widely used in treatment of HCC to prolong survival time, especially when tumors are not surgically respectable [3]. The rationale for TACE is based on the embolization of tumor vessels which predominantly supplied by hepatic arterial blood. Conventional TACE uses an emulsion of Lipiodol-chemotherapeutic agent, whereas TACE with drug-eluting beads (DEB-TACE) uses beads loaded with a chemotherapeutic agent such as doxorubicin. Both two regimens have been shown to achieve a significant survival benefit according to previous researches [4, 5].

Acute lung injury (ALI)/acute respiratory distress syndrome (ARDS) is a type of respiratory failure characterized as the acutely development, bilateral pulmonary infiltrates and severe hypoxemia. ALI/ARDS could be caused by varied etiology, including sepsis, pancreatitis, trauma, pneumonia, and aspiration. Its death rate can reach to 35-50% [6]. Recently, as the prevalence of TACE, increasing clinical cases about post-TACE pulmonary complication had been reported and vast majority resulted in disastrous consequences [7–11]. Now, it is gradually accepted that ALI/ARDS caused by TACE is a rare but fatal complication, mainly thought to be related to chemical injury subsequent to the migration of the infused Lipiodol or chemotherapeutic agent to the lung vasculature. However, so far, most of our knowledge about this complication is based on case reports. Its clinical characteristics and outcomes have not been fully investigated by researchers.

Thus, this study aimed at retrospectively analyzing the common risk factors, clinical features, imaging findings, treatments, and outcomes of TACE-associated ALI. This information will be useful for precise evaluation, early recognition, and management in clinical practice.

## 2. Materials and Methods

### 2.1. Ethics Statement

The institutional ethical committee approved our research protocol. All the patients or their relatives provided written informed consent and understood that their hospital data would be used for research.

### 2.2. Study Patients and Diagnostic Criteria

This study was a retrospective analysis conducted at the First Affiliated Hospital of Zhejiang University. 14 HCC patients with TACE-associated ALI, diagnosed from January 2015 to June 2018, were retrospectively analyzed. Patients, who developed pulmonary complication but no evidence of ALI, were selected as control group. Information was obtained regarding the following clinical parameters: demographic data, symptom, laboratory examination, radiographic presentation, treatment, follow-up, and outcome.

Diagnosis of HCC was made by pathological confirmation (postoperative pathological test or needle biopsy) and typical radiographic evidence (significantly enhanced tumor in the arterial phase and rapidly cleared contrast agents in the portal venous phase). Pulmonary complication defined as the presence of respiratory symptoms (such as cough, sputum, and dyspnea) and abnormalities in chest imaging after TACE. ALI was confirmed according to the standard American-European Consensus Conference (AECC) definition as the development of acute, bilateral pulmonary infiltrates and hypoxemia (ALI: SpO_2_/FiO_2_ < 300; ADRS: SpO_2_/FiO_2_ < 200) in the absence of clinical signs of the left atrial hypertension as the main explanation for pulmonary edema.

### 2.3. TACE Therapy

Puncture with the Seldinger technique was routinely performed with a 5 F catheter being placed into the celiac aorta. Further catheterization was performed in the feeding artery of the intrahepatic tumor after angiography. Next, the intrahepatic tumor was treated with TACE therapy in which iodized oil emulsion or drug-eluting beads loaded with a chemotherapeutic agent were injected into the tumor artery before 300-500 *μ*m particles to completely embolize the tumor feeding artery.

### 2.4. Laboratory Parameters, Treatments, and Outcome Assessments

Once acute respiratory symptom developed after TACE, patient's laboratory examinations including blood gas analysis, CRP, D-dimer, myocardium zymogram level, and sputum or blood culture were tested according to the manufacturer's instructions. Chest computed tomography (CT) was performed using 16-slice or 64-slice systems (Toshiba Aquilion 16 CT Scanner; Brilliance iCT and 64-channel systems). Two experienced radiologists evaluated the CT images, and consensus was achieved by negotiation.

Patients, diagnosed with TACE-associated ALI, were treated with oxygen therapy, antibiotic therapy, systemic corticosteroids, and respiratory support with mechanical ventilation depending on their etiology and the severity of disease.

### 2.5. Statistical Analysis

Continuous variables were presented as mean ± standard deviation and were performed by Student's *t*-test. Categorical variables were presented as frequencies and percentages (*n* (%)). Fisher's exact test was used to compare categorical variables. The survival curves were calculated by the Kaplan-Meier method. Survival differences were evaluated using log-rank test. *P* values < 0.05 were considered statistically significant. All statistical analyses were performed with SPSS v19.0 for Windows (IBM, USA).

## 3. Results

### 3.1. General Clinical Characteristics

During the study period (January 2015 to June 2018), we identified 32 patients with pulmonary complication after TACE and 14 patients (10 female and 4 male) met the diagnostic criteria of ALI were included. The remaining 18 patients served as control group. The demographic features for those 14 patients were presented in Table 1. Briefly, the mean age of those 14 patients was 60.9 ± 11.7 years (range 41-82 years). Cirrhosis was the most common underlying disease (*n* = 10, 71.4%), followed by respiratory disease (*n* = 7, 50%), diabetes (*n* = 1, 7.1%), and hypertension (*n* = 1, 7.1%). The mean tumor diameter was 8.9 ± 3.6 cm. 10 patients (71.4%) were found to have portal vein tumor thrombus, and hepatic arteriovenous fistula was detected in 6 patients (42.9%). Among the 14 cases, 12 patients received conventional TACE therapy with mean dose of 9.6 ± 4.2 mL infused Lipiodol and another 2 patients received DEB-TACE therapy. Compared to control group, the combination of chronic respiratory disease or hepatic arteriovenous fistula were significantly common in patients with ALI (*P* < 0.05), which could be considered as risk factors for the development of ALI following TACE.

### 3.2. Clinical Features

The clinical features of TACE-associated ALI were presented in Table 2. The mean time of ALI onset after TACE was 2.4 ± 1.6 d. Of the 14 patients, 8 patients (57.1%) developed ARDS. Dyspnea was the most common presenting symptom, occurring in 13 patients (92.9%), followed by cough (*n* = 10, 71.4%), fever (*n* = 4, 28.6%), hemoptysis (*n* = 2, 14.3%), and chest pain (*n* = 1, 7.1%). An increase in D-dimer was observed in most of the patients. Blood culture was performed in 4 febrile patients. Klebsiella pneumoniae was isolated from blood culture in 2 patients with an obvious increase in inflammatory markers such as C-reactive protein and white blood cell. The sputum and blood culture in other patients were negative.

**Figure 1:**
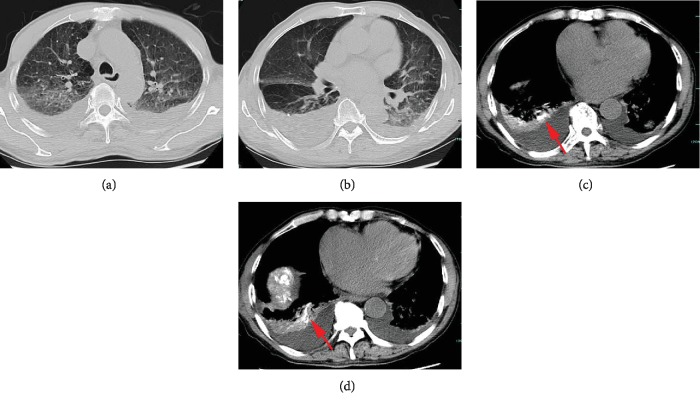
An 82-year-old man with HCC developed ARDS 24 h after the performance of TACE. The chest CT demonstrated diffuse pulmonary infiltration and bilateral pleural effusion (a, b). In addition, an obvious accumulation of Lipiodol (red arrows) was observed in the right lung lobe (c, d).

**Figure 2:**
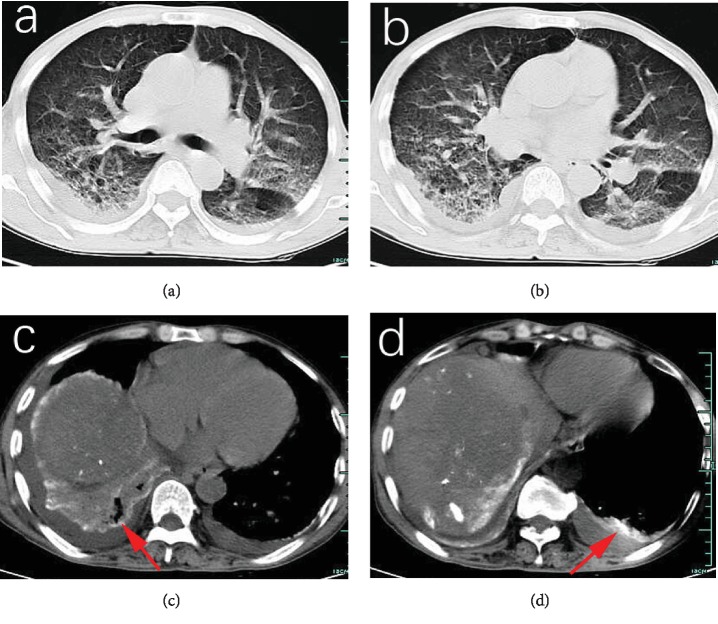
Chest CT findings in a 60-year-old man with an underlying disease of COPD. He suffered from dyspnea and hemoptysis 10 h after TACE for HCC. Chest CT showed diffuse pulmonary infiltration in bilateral lung fields (a, b) and accumulation of Lipiodol (red arrows) located in bilateral lung lower lobes (c, d).

All patients received chest CT scans after the onset of respiratory symptom. Pleural effusion was a frequent finding, which was detected in 9 cases (64.3%). The diffuse pulmonary infiltration (Figures 1(a), 1(b), 2(a), and 2(b)) and accumulation of Lipiodol in lung field (Figures 1(c), 1(d), 2(c), and 2(d)) were regarded as relatively specific findings, which are observed in 6 patients (42.9%). Other associated findings included multiple pulmonary consolidations and ground-glass opacity occurred in 35.7% and 21.4% of the patients, respectively.

### 3.3. Etiology, Treatments, and Outcomes

In current research, we concluded that pulmonary Lipiodol embolization might be one of the main causes of TACE-induced ALI since 6 patients (42.9%) presented with diffuse pulmonary infiltration and obvious accumulation of Lipiodol on chest CT with a negative result in sputum or blood culture. In addition, patients whose blood culture detected Klebsiella pneumoniae were finally diagnosed as having liver abscess and bloodstream infection. Hence, sepsis induced by TACE ought to be another important reason for ALI. However, causes for the remaining 6 cases were unclear.

After a diagnosis of TACE-associated ALI was established, all the patients received oxygen therapy immediately. Other combined treatments including empirical antibiotic therapy (85.7%) and corticosteroids (42.9%) were listed in Table 2. Due to exacerbation of severe hypoxemia, 2 patients were referred to intensive care unit (ICU) for mechanical ventilation and finally died of multiple organ failure or bloodstream infection two weeks later. Another patient with ARDS refused to invasive ventilation and transferred to local hospital, resulting in a death of respiratory failure. Overall, 11 patients achieved remission after treatment and the 30-day mortality rate of TACE-associated ALI was approximately 21.4%.

**Figure 3:**
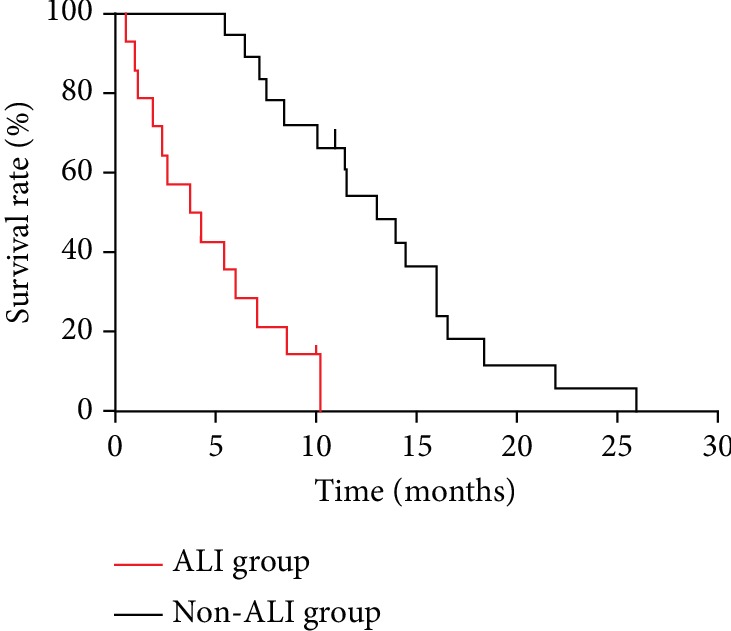
Survival curve of hepatocellular carcinoma patients with TACE-associated pulmonary complications. In ALI group, patient's median survival time was 4.3 months, which was obviously worse than non-ALI group (4.3 months vs. 13.5 months, *P* < 0.05).

Long-term follow-up was available for survivors (Table 3). Due to deterioration of physical condition, merely 4 patients (36.4%) were able to withstand further antitumor therapy including TACE or surgery, while other 7 patients (63.6%) were given best supportive care. The proportion of antitumor therapy in ALI group was significantly lower than that in control group (36.4% vs. 83.3%, *P* < 0.05). Accordingly, the patient's median survival time after the development of ALI was only 4.3 months, which was obviously worse than non-ALI group (4.3 months vs. 13.5 months, *P* < 0.05) (Figure 3). It suggested that the development of ALI could significantly impair the long-term survival of the patients with HCC.

## 4. Discussion

TACE is a widely accepted palliative treatment for patients with advanced HCC. Despite the great advantages of being less invasive and relatively safe, TACE still has multiple side effects [12, 13]. In the present research, we retrospectively analyzed 14 HCC patients who were diagnosed with TACE-associated ALI, revealing a high mortality rate (21.4%) and severe impairment of patient's long-term survival. We found that HCC patients with combined chronic respiratory disease or hepatic arteriovenous fistula were more prone to develop ALI after TACE. Vast majority of patients presented dyspnea, elevated D-dimer, diffuse pulmonary infiltration, and accumulation of Lipiodol on chest CT. Hence, we considered that pulmonary Lipiodol embolism was one of the main causes of TACE-associated ALI. Nevertheless, this study still had some potential limitations. It was a retrospective analysis conducted in a single medical center. The number of ALI cases was relatively small, so the result required further confirmation by large sample clinical studies. In addition, etiological conclusions mainly based on the clinical data analysis, lacking of pathological support.

To date, the mechanisms underlying symptomatic pulmonary injury associated with TACE are not well understood. The most likely mechanism is chemical injury caused by infused Lipiodol or administered medicine. Generally, the procedure of TACE involves mechanical occlusion of selective hepatic artery, supplying HCC with emulsion of Lipiodol-chemotherapeutic agent or beads loaded with a chemotherapeutic agent such as doxorubicin. Early studies had observed that ^131^I-labeled Lipiodol could be detected in the lung when delivered to the hepatic artery of patients with hepatic cancer [14, 15]. Now, increasing evidence supports that pulmonary oil embolism is closely related to the development of ALI/ARDS [16, 17]. Hatamaru et al. reported a case of TACE-associated ARDS due to pulmonary Lipiodol embolism, which confirmed the presence of fat droplets in the pulmonary arteriolar lumen via pathological autopsy [16]. In current study, nearly half of the cases presented diffuse pulmonary infiltration and accumulation of Lipiodol on chest CT but without evidence of infection, which were consistent with findings from other studies [18]. Concerning the underlying molecular mechanisms, it attributed to enzymatic digestion of Lipiodol by lipase and formation of free fatty acid, which is toxic to alveolar-capillary membrane. To date, several risk factors including a large hypervascular HCC (>10 cm) with AV shunts, large-volume Lipiodol infusion (>14.5 mL), and transinferior phrenic artery embolization have been identified for pulmonary Lipiodol embolism after TACE [13, 19, 20]. Thus, recommendations suggest that the maximum safe Lipiodol dose is about 15 to 20 mL or approximately 0.25 mL/kg total body weight.

Although extremely rare, other chemotherapeutic agent may also possess potential for induction of ALI/ARDS when applies in TACE. Recently, acute lung injury following TACE of doxorubicin-loaded LC beads was reported by Khan et al. [8]. Kumasawa et al. described a TACE case using miriplatin with agents developed ARDS 5 days following therapy, presenting as cough, dyspnea, and pulmonary infiltration in chest imaging [7]. In our study, the definite causes of 6 cases were still unclear. We speculated that the etiology of some cases was implicated to chemotherapeutic drug-induced lung injury. In addition, our study revealed 2 ALI patients caused by bloodstream infection, indicating that sepsis secondary to TACE should not be completely ignored, especially in patient with fever and an obvious increase of inflammatory markers.

The optimal management strategy for TACE-associated ALI remains unknown. Oxygenation, systemic corticosteroids, and lung protective ventilation are the primary options and treatments varied according to etiological analysis and the severity of symptoms. Intravenous methylprednisolone might be effective to reduce the inflammatory response, oxidative stress involved in ARDS, and chemical pneumonitis induced by chemotherapeutic agent. It achieved success in 6 cases presented in our research. Nevertheless, the 30-day mortality rate of TACE-associated ALI was still as high as 21.4%. For there are no proven effective therapies, its prevention is quite essential. Precise evaluation, early recognition, and management are critically important during TACE.

In this study, patient's median survival time after the development of ALI was merely 4.3 months, which was significantly worse than non-ALI group. We believed that it closely related to lack of effective antineoplastic therapy since only 36.4% of the patients in ALI group were able to continue further antitumor therapy while the proportion in control group can reach to 83.3%. We inferred that the development of ALI accelerated deterioration of physical condition, leading to a great difficulty for achievement of further antitumor treatment, finally resulting in a poor prognosis.

In summary, this study characterized clinical features and outcomes of ALI caused by TACE, presenting as a lower occurrence rate but a higher mortality. Moreover, the development of ALI could significantly impair the long-term survival of the patients with HCC. Thus, physicians should be fully aware of and avoid this potential fatal complication during TACE.

## Figures and Tables

**Table 1:** Demographic and baseline characteristics of patients with HCC.

Clinical characteristics	ALI (*n* = 14)	Non-ALI^#^ (*n* = 18)	*P* value^∗^
Mean age (years)	60.9 ± 11.7	55.7 ± 5.9	0.1112
Sex, *n* (%)			0.7035
Male	10 (71.4)	14 (77.8)	
Female	4 (28.6)	4 (22.2)	
Cause of HCC, *n* (%)			1.000
Hepatitis B virus	13 (92.9)	16 (88.9)	
Alcoholic liver disease	1 (7.1)	2 (11.1)	
Underlying disease, *n* (%)			
Cirrhosis	10 (71.4)	12 (66.7)	0.4192
Chronic respiratory disease	7 (50)	2 (11.1)	0.0225
Diabetes	1 (7.1)	2 (11.1)	
Hypertension	1 (7.1)	3 (16.7)	
Child-Pugh classification, *n* (%)			1.000
Child class A	8 (57.1)	11 (61.1)	
Child class B	6 (42.9)	7 (38.9)	
Mean tumor diameter (cm)	8.9 ± 3.6	8.0 ± 3.3	0.4677
Portal vein tumor thrombus, *n* (%)			1.000
Presence	10 (71.4)	13 (72.2)	
Absence	4 (28.6)	5 (27.8)	
Hepatic arteriovenous fistula, *n* (%)			0.0265
Presence	6 (42.9)	1 (5.6)	
Absence	8 (57.1)	17 (94.4)	
Therapeutic regimen			1.000
Conventional TACE	13	16	
DEB-TACE	1	2	
Mean Lipiodol dose (mL)	9.6 ± 4.2	8.5 ± 3.6	0.4651

^#^Patients with pulmonary complication but no evidence of ALI; ^∗^significance level set at *P* < 0.05.

**Table 2:** The clinical characteristics of HCC patients with TACE-associated ALI.

Clinical characteristics	No. of patients	Proportion (%)
Mean time to onset (days)	2.4 ± 1.6	
Clinical symptom		
Dyspnea	13	92.9
Cough	10	71.4
Fever	4	28.4
Hemoptysis	2	14.3
Chest pain	1	7.1
Laboratory examination		
ALI (SpO_2_/FiO_2_: 200-300)	6	42.9
ARDS (SpO_2_/FiO_2_: <200)	8	57.1
CRP (mg/L)	79.8 ± 54.2	
D-dimer (*μ*g/mL)	8032 ± 4631	
Positive blood culture	2	14.3
Radiographic finding		
Pleural effusion	9	64.3
Diffuse pulmonary infiltration	6	42.9
Accumulation of Lipiodol in lung field	6	42.9
Multiple pulmonary consolidations	5	35.7
Multiple ground-glass opacity	3	21.4
Possible etiology		
Pulmonary Lipiodol embolization	6	42.9
Sepsis	2	14.3
Unknown	6	42.9
Treatment		
Oxygenation	14	100
Empirical antibiotic therapy	12	85.7
Systemic corticosteroids	6	42.9
Mechanical ventilation	2	14.3

**Table 3:** The outcomes of HCC patients with TACE-associated ALI.

	ALI (*n* = 14)	Non-ALI (*n* = 18)	*P* value^∗^
30-day mortality rate, *n* (%)	3 (21.4)	0 (0)	
Cause of death			
Multiple organ failure	1 (7.1)		
Bloodstream infection	1 (7.1)		
Respiratory failure	1 (7.1)		
Antitumor treatment after remission, *n* (%)			0.0169
TACE and surgery	4 (36.4)	15 (83.3)	
Best supportive care	7 (63.6)	3 (16.7)	
Median survival time after ALI (months)	4.3	13.5	0.0215

^∗^Significance level set at *P* < 0.05.

## Data Availability

The data used to support the findings of this study are included within the article.
